# Circadian modulation of light-evoked avoidance/attraction behavior in *Drosophila*

**DOI:** 10.1371/journal.pone.0201927

**Published:** 2018-08-14

**Authors:** Lisa Soyeon Baik, Yocelyn Recinos, Joshua A. Chevez, Todd C. Holmes

**Affiliations:** Department of Physiology and Biophysics, School of Medicine, University of California at Irvine, Irvine, California, United States of America; McGill University, CANADA

## Abstract

Many insects show strong behavioral responses to short wavelength light. *Drosophila melanogaster* exhibit Cryptochrome- and Hyperkinetic-dependent blue and ultraviolet (UV) light avoidance responses that vary by time-of-day, suggesting that these key sensory behaviors are circadian regulated. Here we show mutant flies lacking core clock genes exhibit defects in both time-of-day responses and valence of UV light avoidance/attraction behavior. Non-genetic environmental disruption of the circadian clock by constant UV light exposure leads to complete loss of rhythmic UV light avoidance/attraction behavior. Flies with ablated or electrically silenced circadian lateral ventral neurons have attenuated avoidance response to UV light. We conclude that circadian clock proteins and the circadian lateral ventral neurons regulate both the timing and the valence of UV light avoidance/attraction. These results provide mechanistic support for Pittendrigh's "escape from light" hypothesis regarding the co-evolution of phototransduction and circadian systems.

## Introduction

The ability to anticipate and adapt to daily environmental changes is critical for survival. In many insects, rhythmic short wavelength light avoidance is crucial for avoiding heat, low humidity, and peak ultraviolet (UV) radiation at midday and thus minimizes a range of hazards from desiccation at organism level to DNA damage at molecular level. This is important particularly for ectotherms like *Drosophila* that maintain their body temperature by behavioral adaptation [1]. Pittendrigh proposed that before the development of Earth’s oxygen rich atmosphere that blocks UV light, that evolution of circadian systems was driven by the need to escape from the harmful effects of UV radiation. CRYPTOCHROME (CRY), the primary circadian light sensor in *Drosophila*, evolved from ancient short wavelength light-activated DNA repair enzymes. We set out to test whether *Drosophila* long-term (hours) behavioral UV light responses are circadian regulated based on an earlier observation that wild-type *Drosophila* exhibit a peak of UV light avoidance behavior at midday under conditions of constant UV light intensity. Peak of UV avoidance in midday coincides with siesta rest in adult flies and flies show preference sleeping in dark environment over light environment during sleep [2]. The peak of midday avoidance coincides with peak UV light intensity in natural environments [3]. Earlier work shows larval acute (minutes) light avoidance behavior depends in part on subsets of circadian pacemaker neurons and circadian genes [4,5].

In *Drosophila*, the short wavelength light-sensitive flavoprotein CRY mediates acute arousal and rapid positive phototaxis responses [6,7]. CRY also mediates temporally slower circadian entrainment and adult light avoidance behavior responses to short wavelength light [3,8,9]. These behavioral responses correspond biophysically to the absorbance spectra of CRY in its baseline flavin adenine dinucleotide (FAD) oxidized state with two major peaks at 365 nm (UV) and 450 nm (blue) [10–12]. Activated CRY mediates blue and UV light-evoked changes in electrophysiological action potential firing rate and resting membrane potential in lateral ventral circadian neurons (LNv) coupled by a voltage-gated potassium beta subunit (Kvβ) called HYPERKINETIC (HK) [3,6,7,13]. HK is a redox sensor that translates redox biochemical signals into changes in membrane electrical potential [3,6,14,15].

In the *Drosophila* brain, approximately 150 pacemaker neurons of the circadian circuit are defined by their expression of ~24 hr cycling PER and TIM [16], of which approximately half express CRY, including the pigment dispersing factor (PDF)-positive lateral ventral neurons (LNv) [17,18]. LNvs show the most rapid white light responses among circadian neurons measured by *period-luciferase* whole circuit dynamic imaging [19]. Clock neuron electrical firing is circadian regulated and their electrical activity drives behaviors [13,20–22]. Together, these findings suggest that light-evoked behaviors may be under circadian regulation.

## Results

### The circadian clock modulates both the valence and the time-of-day dependent changes of UV light avoidance/attraction behavior

**Fig 1:**
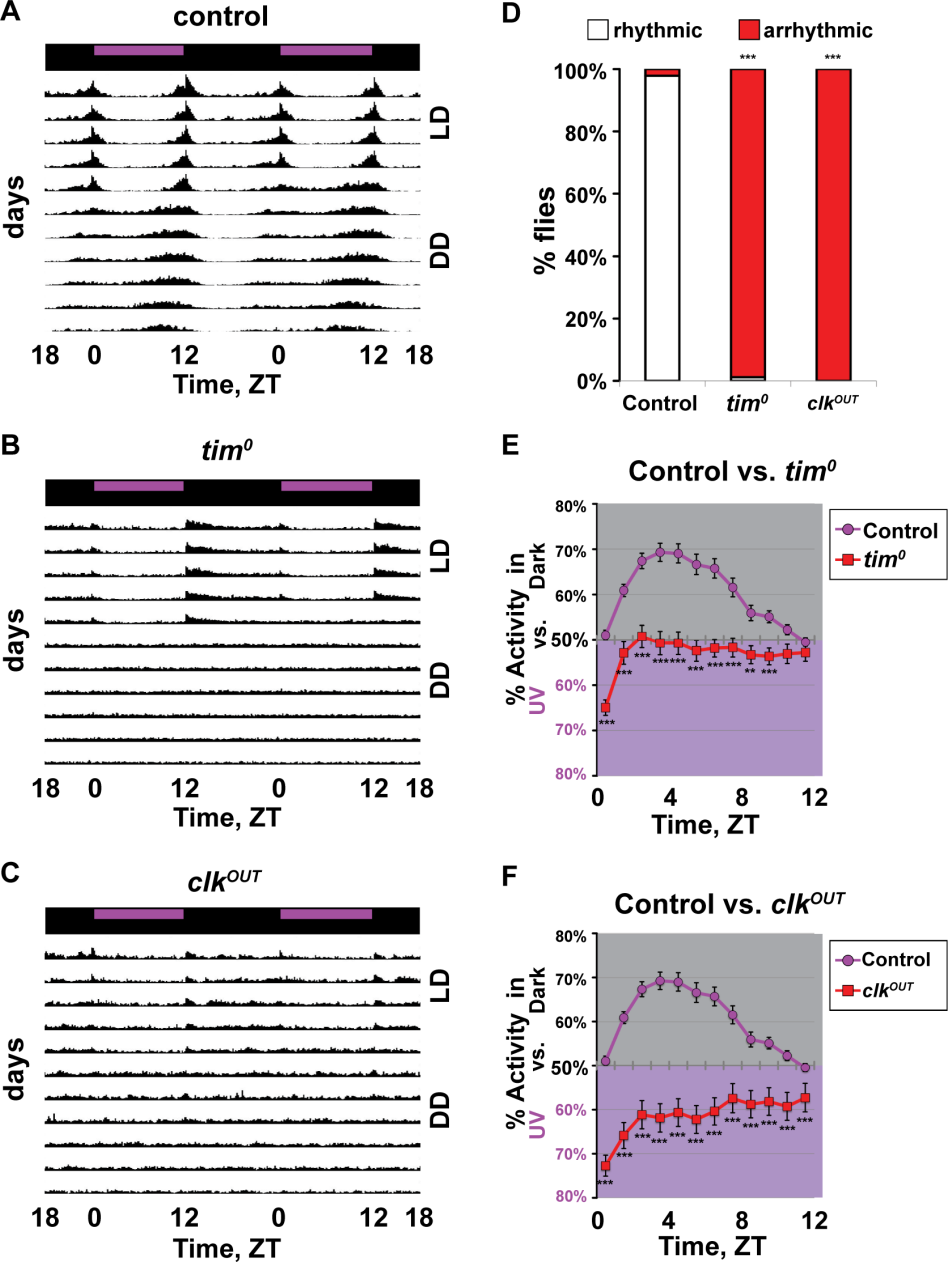
Circadian mutants have defective timing of UV light avoidance behavior. (A-C) Representative double plotted actogram in standard 12h:12h UV (365 nm, 400 μW/cm^2^) light: dark (LD) followed by constant dark condition (DD). (A) Control (*w*^*1118*^; n = 32 flies) flies have normal entrainment in LD and maintains rhythmicity in DD condition. Circadian mutants (B) *tim*^*0*^ (n = 30 flies) and (C) *clk*^*OUT*^ (n = 30 flies) on the other hand show defective entrainment in LD and are arrhythmic in DD. (D) Percentages of rhythmic and arrhythmic flies in DD (control, n = 95 flies; *tim*^*0*^, n = 89 flies; *clk*^*OUT*^, n = 60 flies). (E-F) UV avoidance behavior measured by preference for shaded environment vs. UV-exposed environment (365 nm, 400 μW/cm^2^) calculated by percent of activity in each environment over total activity for each ZT. (E) *tim*^*0*^ flies (n = 71 flies) show significant attenuation of avoidance and defective time-of-day dependent modulation of UV avoidance. (F) *clk*^*OUT*^ flies (n = 66 flies) show a significant valence shift from UV avoidance to strong UV attraction and defective time-of-day dependent modulation of UV avoidance. Data are represented as mean ± S.E.M. *p < 0.05; **p < 0.01; ***p < 0.001 vs. control. See also S1 Fig.

**Fig 2:**
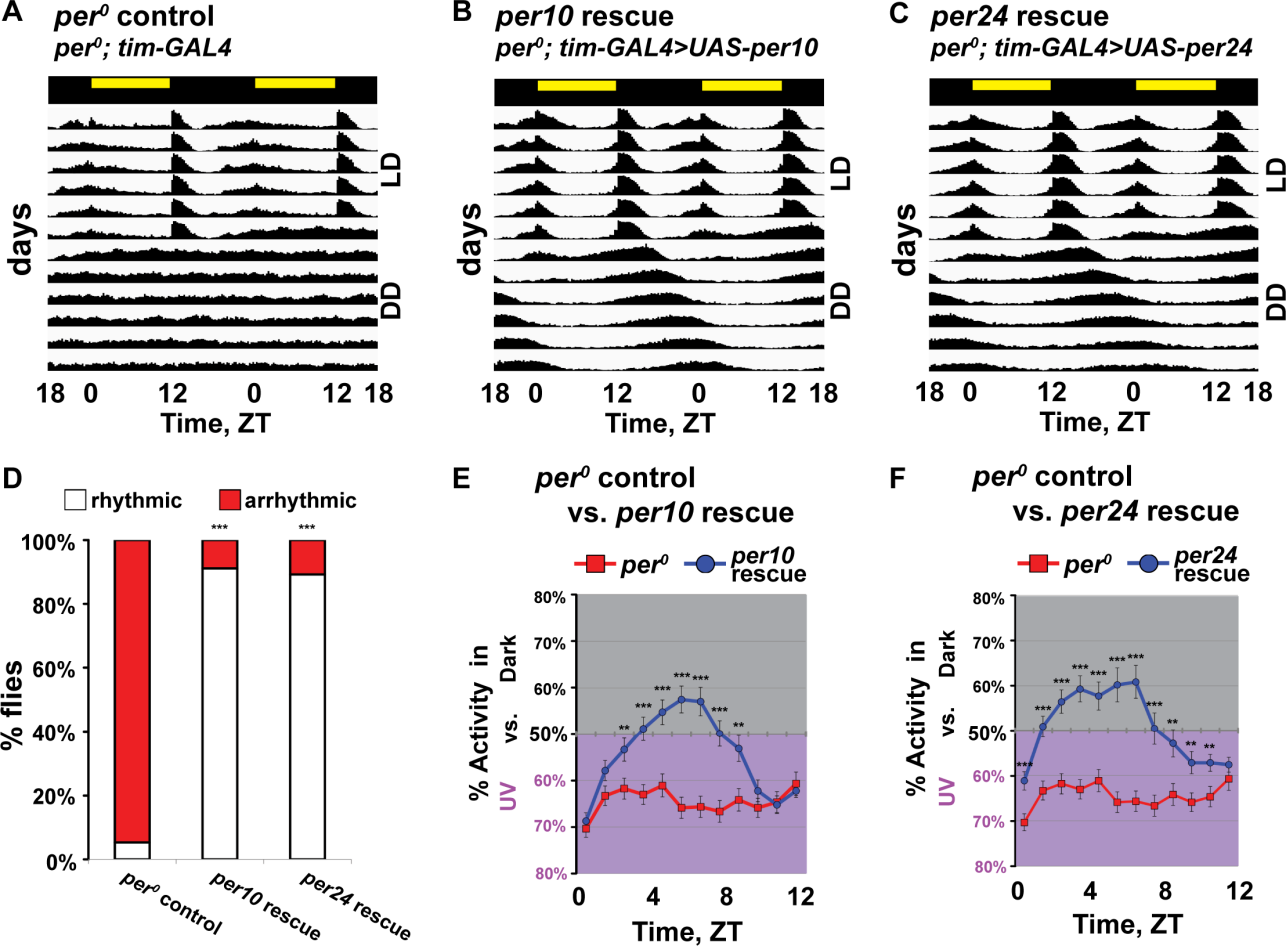
Period expression rescues timing of UV light avoidance behavior. (A-C) Representative double plotted actogram in standard 12h:12h white light: dark (LD) followed by constant dark condition (DD). (A) *Period-null* (driver-only) control (*per*^*0*^; *tim62-GAL4;* n = 32 flies) flies have defective entrainment in LD and are arrhythmic in DD. Flies with tim-GAL4 driven expression of *per* in *per-null* genetic background, (B) per10 rescue (*per*^*0*^; *tim62-GAL4 / UAS-per10*; n = 32 flies) and (C) per24 rescue (*per*^*0*^; *tim62-GAL4 / UAS-per24*; n = 32 flies) show normal entrainment and maintains rhythmicity in DD. (D) Percentages of rhythmic and arrhythmic flies in DD (*Period-null* driver-only control, n = 96 flies; per10 rescue, n = 160 flies; per24 rescue, n = 160 flies). (E-F) UV avoidance behavior measured by preference for shaded environment vs. UV-exposed environment (365 nm, 400 μW/cm^2^) calculated by percent of activity in each environment over total activity for each ZT. *Period-null* control flies (n = 69 flies) show significant attenuation of avoidance and defective time-of-day dependent modulation of UV avoidance. (E) per10 rescue (*per*^*0*^; *tim62-GAL4 / UAS-per10*; n = 104 flies) and (F) per24 rescue (*per*^*0*^; *tim62-GAL4 / UAS-per24*; n = 61 flies) flies have midday peak and time-of-day dependent modulation of UV avoidance. Data are represented as mean ± S.E.M. *p < 0.05; **p < 0.01; ***p < 0.001 vs. *period-*null control.

**Fig 3:**
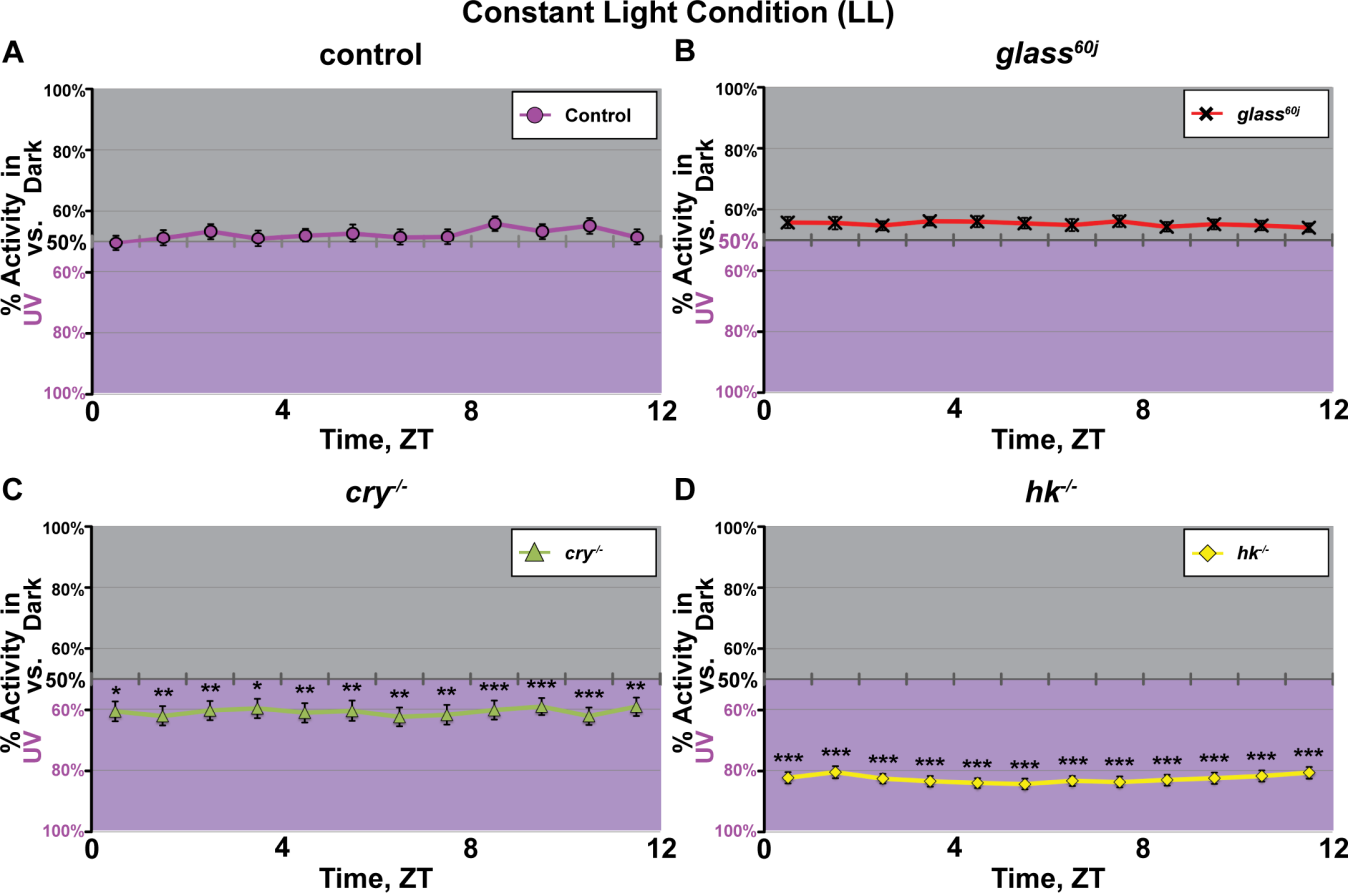
Constant light condition induces valence shifts and eliminates time-of-day dependent modulation of UV light avoidance/attraction behavior. (A-D) UV avoidance/attraction behavior measured by preference for shaded environment vs. UV-exposed environment (365 nm, 400 μW/cm^2^,) calculated by percent of activity in each environment over total activity for each circadian time (CT) in constant light condition (LL). All genotypes tested show valence shifts from UV light avoidance to neutrality or attraction, lack “midday peak” of avoidance behavior, and show no time-dependent modulation in the degree of avoidance/attraction at any time of the day. (A) Control (*w*^*1118*^; n = 45 flies) and (B) *glass*^*60j*^ (n = 46 flies) flies show neutrality or slight avoidance, slightly preferring the shaded environment over the UV-exposed environment (all are not significant compared to control). In comparison, (C) *cry*^*-/-*^ (n = 43 flies) and (D) *hk*^*-/-*^ (n = 46 flies) flies exhibit strong attraction to the UV-exposed environment at all times of the day. Data are represented as mean ± S.E.M. *p < 0.05; **p < 0.01; ***p < 0.001 vs. *w*^*1118*^ control.

To test the hypothesis that adult UV light avoidance behavior is circadian regulated, we measured this behavior in mutant flies lacking core circadian genes. Control *w*^*1118*^ flies show normal entrainment in standard 12h:12h UV light:dark (LD) followed by sustained rhythmic activity in constant darkness (DD) (Fig 1A and 1D, and Panel A and E in S1 Fig). Circadian clock mutants lack rhythmic behavior in DD [23] (Figs 1B–1D, 2A, 2D and S1 Fig). Clock gene null mutants *tim*^*0*^, *clk*^*OUT*^, and *per*^*0*^ (all in the *w*^*1118*^ genetic background) have defects in DD rhythms, entrainment, and LD morning/evening anticipation behavior (Figs 1B–1D, 2A, 2D and Panel B-D and F-H in S1 Fig). These features of anticipatory behavior and rhythmicity of locomotor activity in DD are indicators of a functional clock. Behavioral UV light avoidance in adult control flies is absent in the beginning of the morning, steadily rises and peaks during the midday, and gradually decreases approaching simulated “dusk” (ZT9-12) (Fig 1E; [3]) in response to constant UV light levels throughout the 12hr day. Clock gene null mutants *tim*^*0*^, *clk*^*OUT*^, and *per*^*0*^ flies all show defective time-of-day dependent modulation UV avoidance exhibited by controls (Figs 1E and 1F and 2E and 2F). Circadian mutant *tim*^*0*^ flies not only fail to show the midday UV avoidance peak, but also shift their behavioral valence to strong attraction to the UV light-exposed environment over the shaded environment during the first hour of the day, then subsequently show mostly weak attraction to the UV light-exposed environment after ZT1 (Fig 1E). Mutant *clk*^*OUT*^ and *per*^*0*^ flies exhibit an even stronger valence shift to UV light attraction throughout the entire day along with defective time-of-day dependent modulation of UV avoidance compared to wild-type controls (Figs 1F and 2E and 2F).

To determine whether valence and midday peak of avoidance to UV light along with rhythmicity in DD can be rescued, *tim-GAL4* driven genetic rescue of *per* expression in *per-null* mutant background was tested for light environmental choice assay. We confirmed that *tim-GAL4* driven expression of either of two *UAS-per* lines (*UAS-per10* or *UAS-per24*) in the *per*^*0*^ genetic background rescues normal LD entrainment and rhythmicity in DD (Fig 2B–2D). Unlike *per*^*0*^ driver-only negative control flies, flies with genetic rescue of *per10* or *per24* exhibit time-of-day dependent modulation of UV light avoidance/attraction behavior, including a midday peak of UV light avoidance followed by a gradual decrease approaching simulated “dusk” (ZT9-12) (Fig 2E and 2F). The valence for UV avoidance is rescued in the midday but does not fully rescue to the wild-type control level (Figs 1 and 2E and 2F). Together, our results show that the molecular circadian clock modulates time-of-day dependent changes of UV light avoidance/attraction behavior to elicit a peak of UV avoidance in the midday.

### Constant light-induced disruption of the circadian clock abolishes time-of-day dependent changes of UV light avoidance behavior and reveals that CRY and HK regulate the valence of UV light avoidance and attraction

Constant light (LL) disrupts the circadian clock in many wild-type animals [24,25]. Mutant *cry*^*-/-*^ flies’ locomotor activity remain rhythmic in LL (S2 Fig) [26,27]. Thus, exposure to LL provides an environmental means to render the clock arrhythmic without the use of genetic mutants. We tested wild-type flies for UV light (365 nm, 400 μW/cm^2^) avoidance/attraction under LL using the light choice assay[3], along with LL-exposed mutant flies that lack molecular and structural components of light input pathways (*cry*^*-/-*^, *hk*^*-/-*^, and *glass*^*60j*^). All flies tested share the *w*^*1118*^ genetic background. In the circadian-disrupting LL condition, all flies completely lack time-of-day dependent changes in UV avoidance/attraction behavior (Fig 3). Control wild-type flies exposed to LL lack not only time-of-day dependent modulation of UV light avoidance and show no preference or weak preference of shade throughout the day as compared to the integrated activity under LD conditions for which the circadian clock is intact (Fig 3A). Similarly, *glass*^*60j*^ flies lack time-of-day dependent modulation of UV light avoidance throughout the daytime and show weak preference of shade throughout the day with no significant differences from control at any time point tested (Fig 3B). In contrast, mutant *cry*^*-/-*^ flies lack time-of-day dependent modulation and show significantly greater preference than control for the high intensity UV-exposed environment at all times tested, consistent with their loss of avoidance in standard LD light choice assay (Fig 3C, and Panel A in S4 Fig). Mutant *hk*^*-/-*^ flies also show significantly greater preference than control to UV light at all times (Fig 3D) in contrast to neutral to slight UV avoidance exhibited by control and *glass*^*60j*^ flies (Fig 3A and 3B). The steady value of avoidance/attraction seen for each genotype under LL resembles its trough of avoidance/attraction oscillation seen in LD. The LL-evoked clock disruption eliminates the circadian “filter” that underlies time-of-day dependent modulation of UV light avoidance behavior. When the circadian system is disrupted environmentally via constant light exposure, *cry*^*-/-*^ and *hk*^*-/-*^ mutants show constant level of attraction to UV light-exposed environment at all times of day. This suggests an attraction/avoidance valence that is dependent on CRY/HK, and independent of the circadian function. Together these results suggest the surprising finding that the clock itself by multiple measurements contributes to UV light avoidance/attraction valence and reveals the inhibitory signal produced by the UV light activated CRY/HK pathway that alters the valence between light-evoked avoidance and attraction.

### LNv circadian neurons are necessary for normal UV avoidance behavior

**Fig 4:**
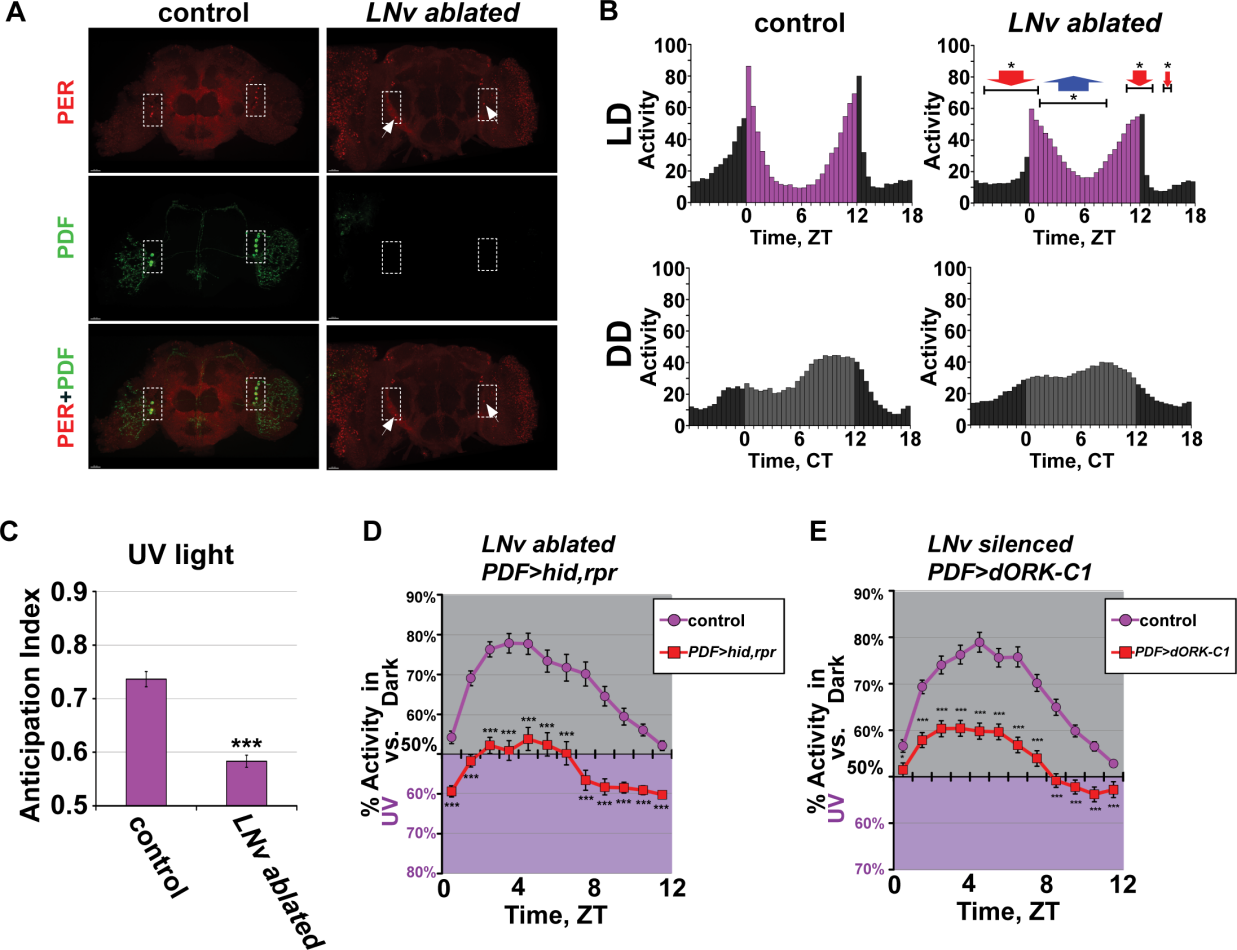
LNv circadian cells modulate the valence of UV light avoidance/attraction behavior but not timing. (A) Control (*UAS-hid*, *rpr; +*; left panels) and LNv ablated (*UAS-hid*, *rpr; pdfGAL4-p12c;* right panels) brains stained with anti-PER (red) and anti-PDF (green) shows successful ablation of PDF-positive LNv neurons (dashed white box) in the LNv ablated flies as evident by the lack of PDF staining, while PDF-negative 5^th^ small-LNv is intact (arrow). Scale bar represents 30μm. (B) Average activity plot of control (*w*^*1118*^; n = 96 flies) (left panels) and LNv ablated flies (*UAS-hid*, *rpr; pdfGAL4-p12c;* n = 285 flies) (right panels) in 12h:12h UV (365 nm, 400 μW/cm^2^) light: dark (LD) (top panels; 5 days) followed by constant darkness (DD) (bottom panels; 5 days). LNv ablated flies (right panels) have defective circadian activity profile in both UV LD and DD conditions (top panels, and bottom panels, respectively). Arrows represent significantly higher (blue arrow, *p<0.05) or significantly lower (red arrow, *p<0.05) average activity in LNv ablated flies compared to control in the represented bin(s) during LD. (C) Harrisingh morning anticipation index (ref. 45) for control (left) versus LNv ablated (*UAS-hid*, *rpr; pdfGAL4-p12c;* right) during LD. LNv ablated flies have significantly lower morning anticipation compared to control during UV (365 nm, 400 μW/cm^2^) light LD (control, n = 95 versus LNv ablated, n = 280, ***p<0.001). (D-E) UV avoidance/attraction behavior measured by preference for shaded environment vs. UV-exposed (365 nm, 400 μW/cm^2^) calculated by percent of activity in each environment over total activity for each ZT. Both (D) LNv ablated flies (*UAS-hid*, *rpr; pdfGAL4-p12c*) and (E) LNv silenced flies (*w; pdfGAL4; UAS-dORK-C1*) have significant defects in UV light avoidance behavior at all times of the day compared to control flies (n = 78 flies, control vs. n = 76 flies, LNv ablated; n = 110 flies, control vs. n = 92 flies, LNv silenced), but maintain time-of-day dependent pattern of modulation in avoidance behavior. Data are represented as mean ± S.E.M. *p < 0.05; ***p < 0.001 vs. control. See also S1 and S2 Figs.

To test whether circadian lateral ventral neurons (LNv) are important for UV light-evoked avoidance behavior, we generated LNv ablated flies by transgenic pdfGAL4-directed dual expression of the cell death genes *head involution defective* (*hid*) and *reaper*. Ablation of LNv neurons is confirmed by immunocytochemistry of whole adult fly brain as shown by the absence of PDF staining. PER staining shows specific absence of PER staining of LNv neurons, with the exception of the 5^th^ small LNvs, which do not express PDF (Fig 4A [28]). PER also stains the lateral dorsal neurons (LNd) and dorsal neurons 1 and 3 (DN1, DN3) in both control and PDF-expressing LNv ablated flies, demonstrating a PDF-positive LNv-specific circadian neuron ablation. We further confirm the ablation of LNv neurons by behavioral analysis of disrupted afternoon and morning activity, phase advanced evening activity, and decreased anticipation (Fig 4B and 4C, and S3 Fig) [29–32]). The slight phase advance of evening activity in LNv ablated flies resembles the phase advance of evening activity seen in *pdf-null* flies [30]. Further, LNv ablated flies show significantly higher midday activity/siesta disruption and lower average locomotor activity in both evening and morning relative to controls under 12h:12h UV LD (Fig 4B). Thus, the LNvs regulate midday behavior as well as evening and morning behavior [29–33].

LNv ablated flies tested with the UV light choice assay show time-of-day dependent changes in avoidance/attraction (Fig 4D, and S4 Fig). Most striking, their UV light response shifts significantly from UV light avoidance to attraction compared to control flies (Fig 4D), which phenocopies the valence shift of UV light attraction seen in *cry*^*-/-*^ and *hk*^*-/-*^ flies (S4 Fig [3]). To test whether membrane excitability of LNvs is important for the timing and/or valence of UV light behavioral responses, we genetically attenuated membrane excitability of LNvs by expressing *Drosophila* open-rectifier K^+^ channel (dORK) specifically in LNv neurons using a *pdf-GAL4* driver [34,35]. Electrically silenced-LNv fly UV light avoidance/attraction behavior is qualitatively similar to LNv ablated flies as well as *cry*^*-/-*^ and *hk*^*-/-*^ flies (Fig 4D and 4E and S4 Fig) as shown by the significant attenuation of UV-evoked avoidance that still peaks at midday (Fig 4E). These results suggest that the LNv, like CRY and HK, contribute more to the regulation of the valence of the UV light response between avoidance and attraction than the time-of-day modulation of UV light avoidance/attraction. We conclude that the LNv circadian neurons and their electrical activity modulate the valence of UV light choice response behavior in adult *Drosophila*.

### The CRY/HK phototransduction pathway modulates the timing and valence of blue and orange light choice behavior

**Fig 5:**
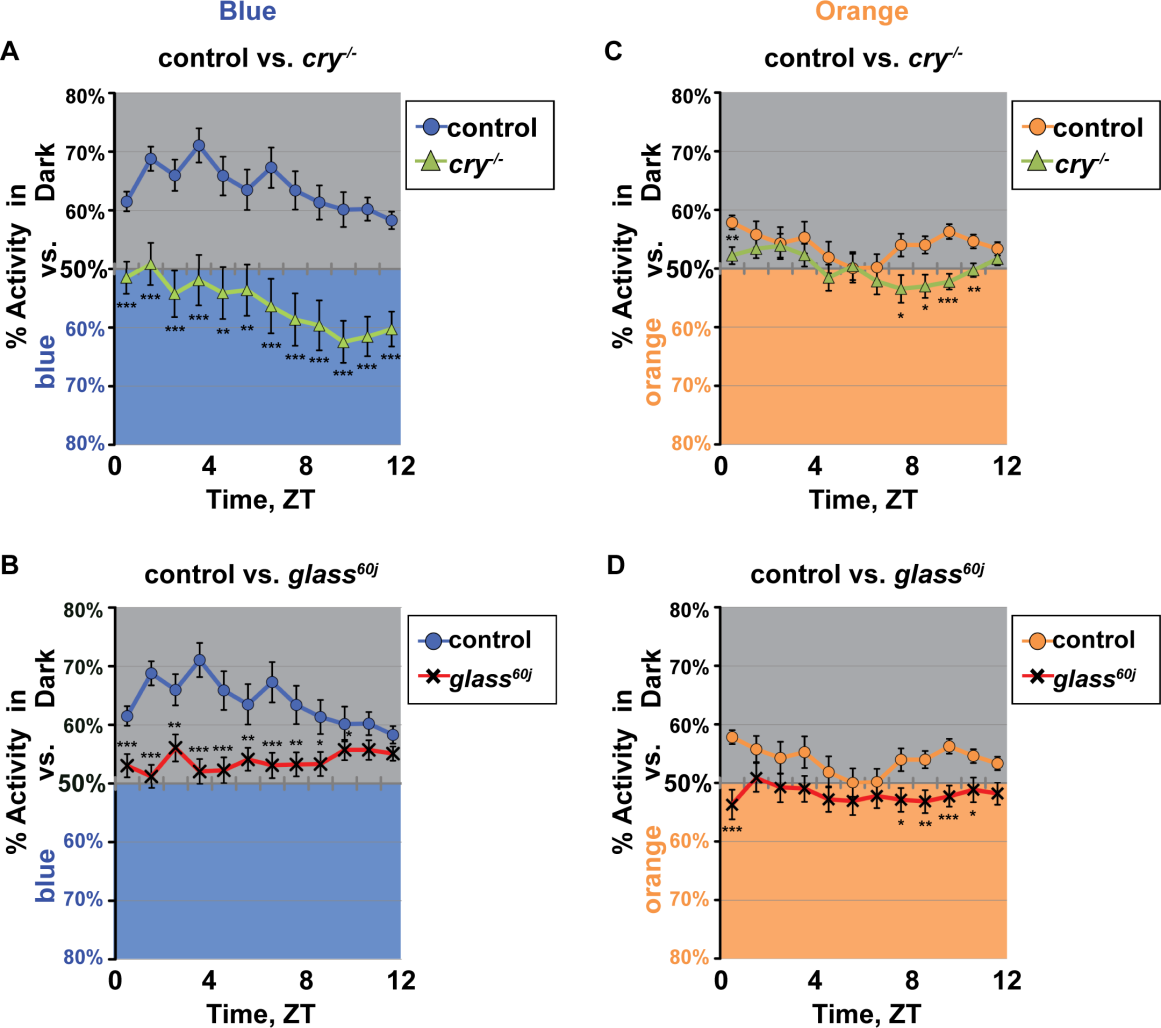
The CRY-mediated phototransduction modulates blue and orange light avoidance/ attraction behavior. (A-B) Blue light avoidance behavior measured by preference by shaded environment vs. blue-light exposed environment (400 μW/cm^2^, 460 nm) calculated by percent of activity in each environment over total activity for each ZT. (A) Control flies (*w*^*1118*^; n = 75 flies) have significant avoidance to blue light with midday peak of avoidance. In contrast, null *cry*^*-/-*^ flies (n = 58 flies) show a significant valence shift from avoidance to attraction for blue light. (B) *glass*^*60j*^ flies (n = 78 flies) avoid blue light at all times of the daytime, but have attenuated response relative to control. (C-D) Orange light avoidance behavior measured by preference by shaded environment vs. orange-light exposed environment (400 μW/cm^2^, 595 nm) calculated by percent of activity in each environment over total activity for each ZT. (C) Control flies (n = 63 flies) and *cry*^*-/-*^ flies (n = 64 flies) show overall neutral attraction/ avoidance responses to orange light, but *cry*^*-/-*^ flies show small but significant valence shifts to attraction. (D) Similarly, *glass*^*60j*^ flies (n = 62 flies) show overall neutral attraction / avoidance to orange light, but *glass*^*60j*^ flies show small but significant valence shifts to attraction. Data are represented as mean ± S.E.M. *p < 0.05; **p < 0.01; ***p < 0.001 vs. control.

Baseline FAD oxidized CRY exhibits a blue light excitation peak at 450 nm in addition to the UV light peak at 365 nm. CRY excitation ceases in the green light spectral range around 525 nm [10–12]. Control flies tested for light choice in response to 450 nm blue light exhibit significant avoidance behavior that is qualitatively similar to UV light avoidance behavior (Fig 5A–5C). Mutant *cry*^*-/-*^ null flies show a significant valence shift to blue light attraction that peaks towards the end of the day (Fig 5A). In contrast, *glass*^*60j*^ mutant flies show mostly neutral behavior with very weak avoidance to blue light (*glass*^*60j*^ flies express CRY) (Fig 5B). Control, *cry*^*-/-*^, and *glass*^*60j*^ flies tested for light choice in response to 595 nm orange light exhibit mostly neutral avoidance/attraction behavior with the exception of a few time points for *cry*^*-/-*^, and *glass*^*60j*^ flies which show very weak light avoidance / attraction to orange light (Fig 5C and 5D).

## Discussion

The circadian clock and circadian circuit neurons modulate both the valence of UV light avoidance/attraction and the timing of rhythmic behavioral responses to high intensity UV light (Figs 1–4). CRY/HK- and opsin-mediated external and internal photoreceptors all contribute to circadian clock entrainment to light [36,37] and modulate complex short wavelength light avoidance/attraction behavior (Figs 3 and 5). The disrupted valence is particularly clear and prominent in the circadian-disrupting LL light condition without the circadian “filtering” on time-of-day dependent modulation for all genotypes including wild-type. In LL, both *cry*^*-/-*^ and *hk*^*-/-*^ flies display a steady attraction to UV light exposed environment at all times of day (Fig 3C and 3D). Together, these results strongly suggest that both the circadian clock and the CRY/HK signaling pathway code for UV light avoidance. Mutant *hk*^*-/-*^ flies exhibit strong behavioral attraction to UV light exposed environments, even more so than *cry*^*-/-*^ flies for both LD and LL light choice assays (Fig 3D, and Panel B in S4 Fig). HK is a functional redox sensor [14,15,38]. It is likely that very high intensity UV light generates redox signals even in the absence of CRY [39,40]. Surprisingly, all circadian mutants tested show degrees of valence shift from avoidance to attraction to the UV light-exposed environment and that this can be rescued by genetic clock restoration (Figs 1E and 1F and 2E and 2F).

In addition to rhythmic UV light avoidance, temperature preference rhythm further refines a complex and adaptive behavioral output in *Drosophila* [41,42]. Certain *Drosophila* sensory responses, including temperature preference, olfactory response, and gustatory response, are under circadian control [42–44]. An afternoon peak (A-peak) of locomotor activity appears under semi-natural conditions with increased temperature or light intensities and likely facilitates escape from harmful environments to shade to avoid desiccation [45,46]. Midday avoidance is coordinated between multiple sensory modalities, as temperature preference is dependent on light environment [47]. Timing of peak midday UV avoidance coincides with siesta rest in adult flies, which prefer to sleep in dark environments [2]. In the absence of environmental choice between UV light exposure and shade, LNv ablated flies exhibit disrupted midday siesta rest with significantly higher locomotor activity along with dampened evening and morning activity relative to controls under 12h:12h UV LD (Fig 4B). CRY also mediates similar valence control over blue light responses (Fig 5). We propose that electrical signaling by LNv neurons and UV light sensing by CRY/HK are crucial in regulating the valence of the UV light response between avoidance and attraction to coordinate the fundamental escape from light [48]. This provides both core circadian clock components and the CRY/HK as coordinating elements for Pittendrigh’s idea of “escape from light” that the circadian systems were evolved to effectively escape the harmful effects of short wavelength light [48].

## Materials and methods

### Fly lines

Fly lines were systemically backcrossed to *w*^*1118*^ for at least six generations. Per rescue flies were generated by crossing female *per*^*9*^*; tim62-GAL4* flies to male *UAS-per10* or *UAS-per24* flies. LNv ablated flies were generated by crossing female *UAS-hid*, *reaper* to male *pdfGAL4* flies, then crossing the males from the F_1_ generation to female *UAS-hid*, *reaper* flies. LNv silenced flies were generated by crossing *pdfGAL4* flies with *UAS-dORKΔ-C1* flies.

### Locomotor analysis

Locomotor activity of individual flies was measured using the TriKinetics Locomotor Activity Monitoring System via infrared beam-crossing recording total crosses in 15 or 30 min bins. Actograms were generated using Clocklab software. Average activity eduction graphs, % rhythmic flies, and its statistics were measured using FaasX software and Microsoft Excel. For UV LD and LL experiments, Philips TL-D Blacklight UV source with narrow peak wavelength of 365 nm and intensity of 400 μW/cm^2^ was used.

### Light choice assay

LD Light choice assays were conducted as outlined in [3]. For constant light (LL) light choice assay, the protocol was modified as follows: when one-half of the monitors were covered, instead of 12h:12h UV light (365 nm, 400 μW/cm^2^):dark, the UV light was constantly left on.

### Immunocytochemistry

Brains were dissected in 1X PBS, fixed in 4% paraformaldehyde (PFA) for 30min, washed 3X 10min in PBS-Triton-X 1%, incubated in blocking buffer (10% Horse Serum-PBS-Triton-X 0.5%) at room temperature before incubation with mouse α-PDF C7, monoclonal (1:10,000) and rabbit α-PER, polyclonal (1:1,000) antibodies overnight in 4°C. Brains were washed 3X 10min in PBS-Triton-X 0.5% then incubated in goat α-mouse-Alexa- (1:500) and goat α-rabbit-Alexa-594 (1:500) secondary antibodies in blocking buffer overnight in 4°C. Brains were washed 5X 15min in PBS-Triton-X 0.5% before mounting in Vectashield mounting media (Vector Laboratories). Microscopy was performed using Zeiss LSM700 confocal microscope.

### Anticipation index

Morning anticipation index was calculated using the Harrisingh/Individual index. For individual fly, fraction of activity during the 3 hours before ZT0 was compared to the activity level through the six hours before ZT0 [49].

## Supporting information

S1 Fig Circadian mutants have disrupted entrainment to UV light. (A-D) Representative average activity plot in standard UV (365 nm, 400 μW/cm^2^) light: dark 12:12 LD (5 days). (A) Control (n = 32 flies) flies entrain to UV light LD, but (B) *per*^*0*^ (n = 30 flies), (C) *tim*^*0*^ (n = 30 flies), and (D) *clk*^*OUT*^ (n = 30 flies) have defective entrainment in LD. (E-H) Average activity plot in constant darkness (DD) (5 days) that followed UV LD. (E) control (n = 32 flies) maintain rhythmicity, but (F) *per*^*0*^ (n = 30 flies), (G) *tim*^*0*^ (n = 30 flies), and (H) *clk*^*OUT*^ (n = 30 flies) flies are arrhythmic in DD.
(PDF)

S2 Fig Constant UV light condition disrupts locomotor activity rhythm. (A-C) Representative double plotted locomotor actogram in 5 days of standard 12h:12h UV (365 nm, 400 μW/cm^2^) light: dark (LD) followed by 6 days of constant UV light condition (LL). (A) Control (*w*^*1118*^; n = 47 flies) flies have normal entrainment in LD and becomes arrhythmic in LL. (B) *glass*^*60j*^ (n = 87 flies) also becomes arrhythmic in LL. (C) *cry*^*-/-*^ (n = 87 flies) on the other hand maintain rhythmicity in UV LL. (D) *hk*^*-/-*^ (n = 90 flies) become arrhythmic in LL.
(E) Percentages of rhythmic and arrhythmic flies in LL. Data are represented as mean ± S.E.M. ***p < 0.001 vs. control.
(PDF)

S3 Fig LNv ablated flies have defects in locomotor activity profile throughout the day. (A-D) Average activity plot of control (n = 96 flies) (top panels) and LNv ablated flies (*UAS-hid*, *rpr; pdfGAL4-p12c;* n = 256 flies) (bottom panels) in standard 12h:12h white light: dark (LD) (left panels; 5 days) followed by constant darkness (DD) (right panels; 5 days). Arrows represent significantly higher (blue arrow, *p<0.05) or significantly lower (red arrow, *p<0.05) average activity in LNv ablated flies compared to control in the represented bin(s) throughout the day during LD. Compared to (A) control flies (n = 96 flies), (C) PDF+ (LNv) ablated flies (*UAS-hid*, *rpr; pdfGAL4-p12c;* n = 256 flies) show defective locomotor activity in LD. (B, D) Average activity plot in constant darkness (DD) (5 days) that followed LD. (B) Control and (D) LNv ablated flies both maintain rhythmicity in DD, but LNv ablated flies show defective locomotor activity in DD compared to control flies. (E) Harrisingh morning anticipation index for control (left) versus LNv ablated (*UAS-hid*, *rpr; pdfGAL4-p12c;* right) during LD. LNv ablated flies have significantly lower morning anticipation compared to control during white light LD (control, n = 64 versus LNv ablated, n = 159, ***p<0.001). Data are represented as mean ± S.E.M. *p < 0.05; ***p < 0.001 vs. control.
(PDF)

S4 Fig LNv -ablated or -silenced flies phenocopy the valence shift from UV light avoidance to attraction seen in *cry*^*-/-*^ *and hk*^*-/-*^ flies. (A-B) UV avoidance behavior measured by preference for shaded environment vs. UV-exposed (365 nm, 400 μW/cm^2^) calculated by percent of activity in each environment over total activity for each ZT. LNv ablated flies (*UAS-hid*, *rpr; pdfGAL4-p12c*; n = 76 flies) closely mimic the time-of-day dependent circadian modulation and valence of UV light avoidance behavior of (A) *cry*^*-/-*^ (n = 78, modified from Baik et al., 2017, *PNAS*) and (B) *hk*^*-/-*^ (n = 77, modified from Baik et al., 2017, *PNAS*) flies. Data are represented as mean ± S.E.M. *p < 0.05; ***p < 0.001.
(PDF)
