# Supplementary figures and images for: Circadian modulation of light-evoked avoidance/attraction behavior in Drosophila
Source: PLoS One. 2018 Aug 14;13(8):e0201927. doi: 10.1371/journal.pone.0201927 (PMC6091921; doi:10.1371/journal.pone.0201927)

**LD****A**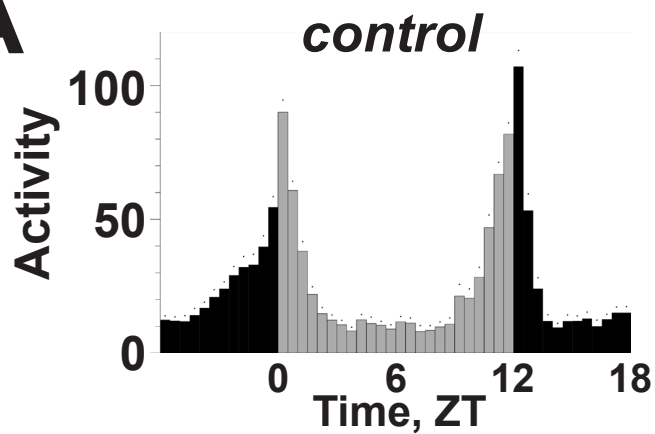**B**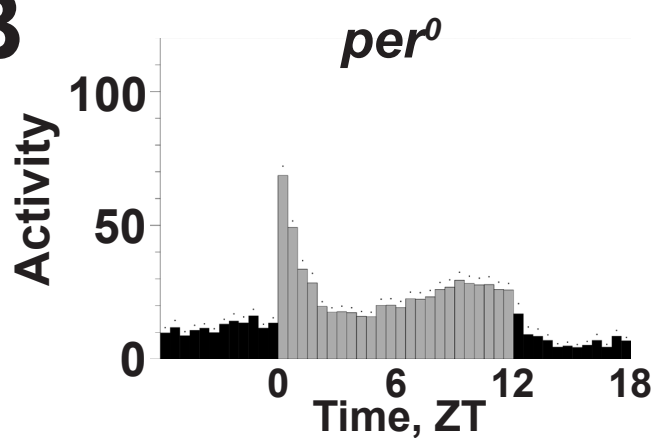**C**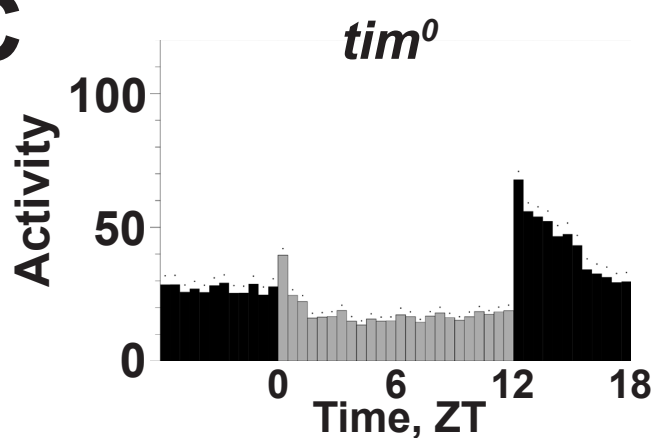**D**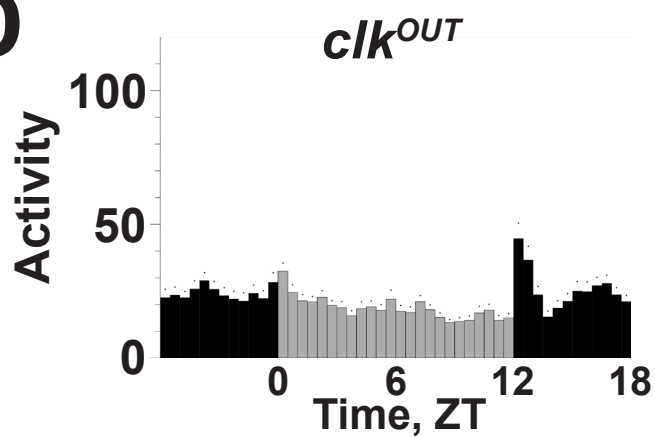**DD****E**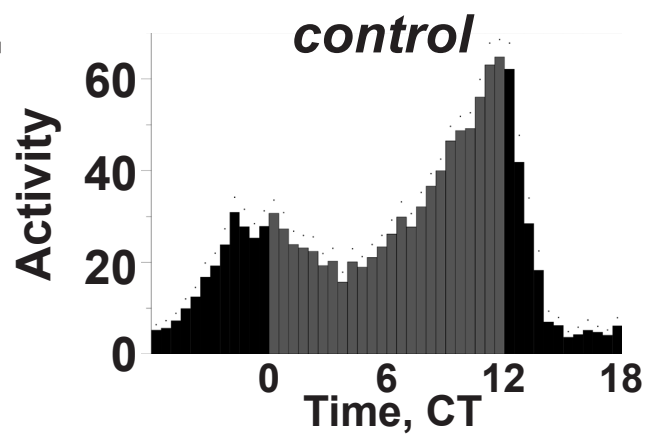**F**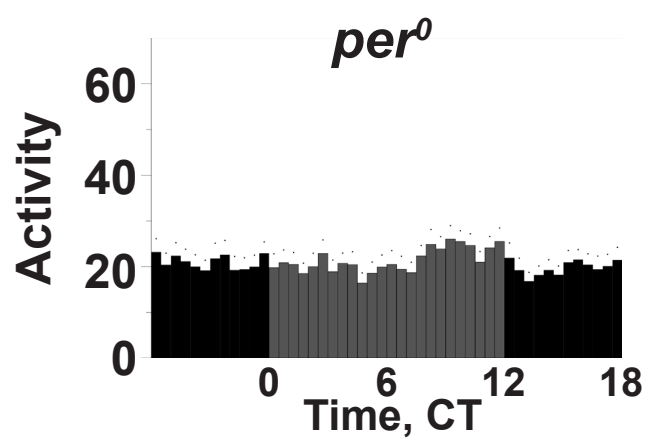**G**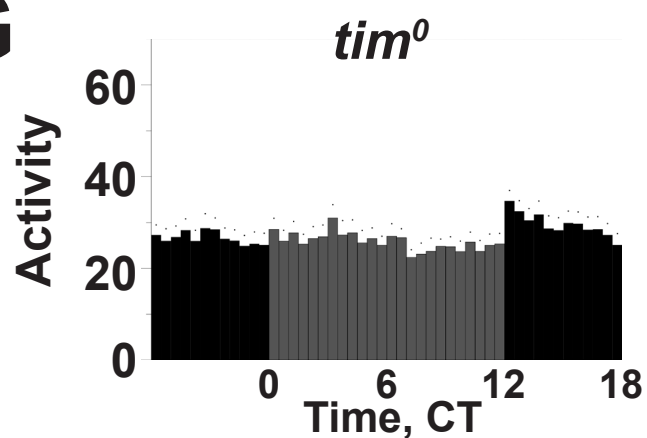**H**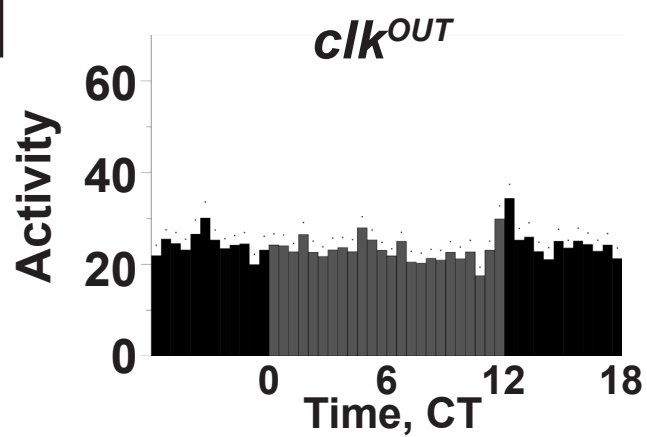

Supplement: S1 Fig — (A-D) Representative average activity plot in standard UV (365 nm, 400 μW/cm2) light: dark 12:12 LD (5 days). (A) Control (n = 32 flies) flies entrain to UV light LD, but (B) per0 (n = 30 flies), (C) tim0 (n = 30 flies), and (D) clkOUT (n = 30 flies) have defective entrainment in LD. (E-H) Average activity plot in constant darkness (DD) (5 days) that followed UV LD. (E) control (n = 32 flies) maintain rhythmicity, but (F) per0 (n = 30 flies), (G) tim0 (n = 30 flies), and (H) clkOUT (n = 30 flies) flies are arrhythmic in DD. (PDF) [file pone.0201927.s001.pdf]

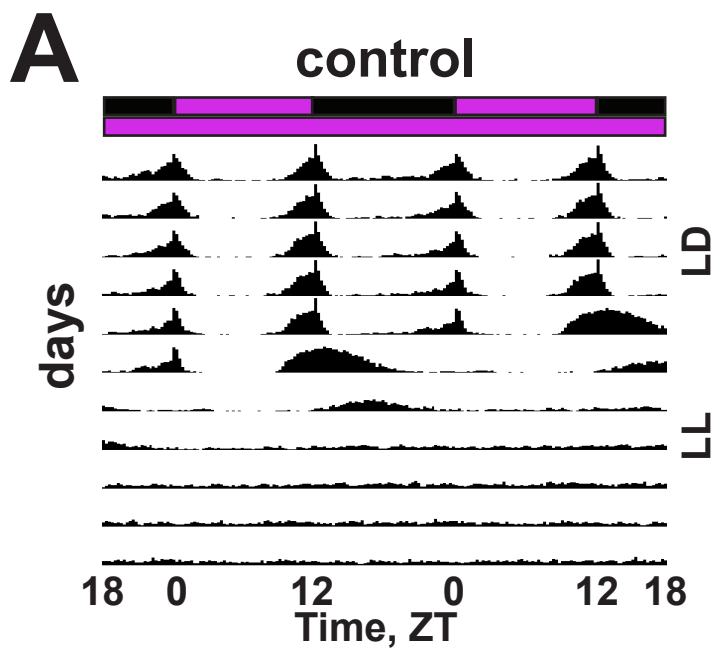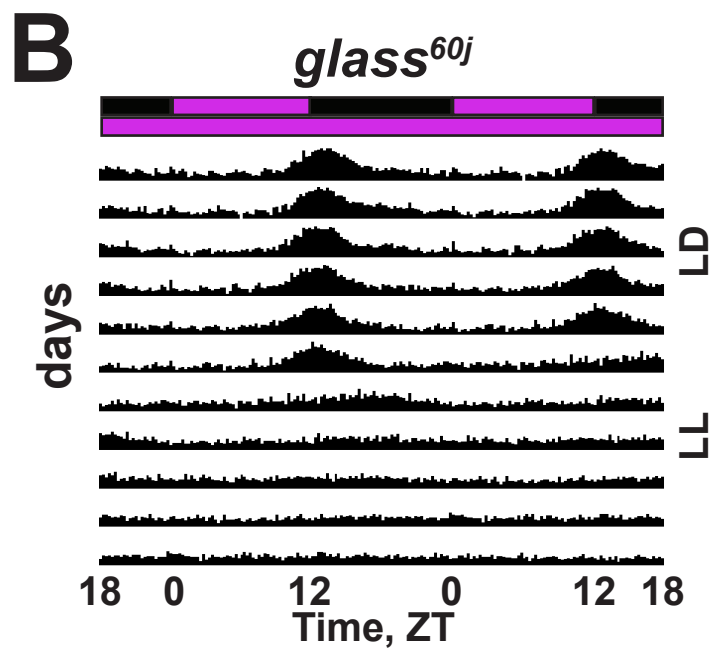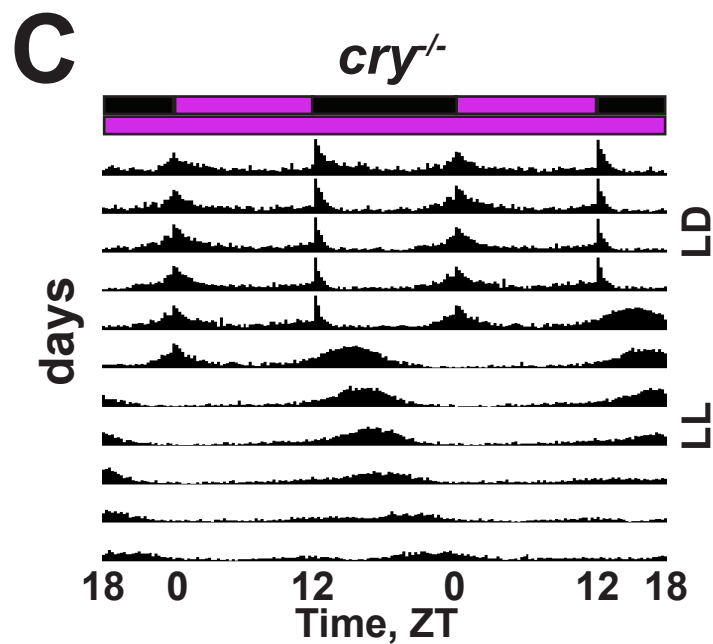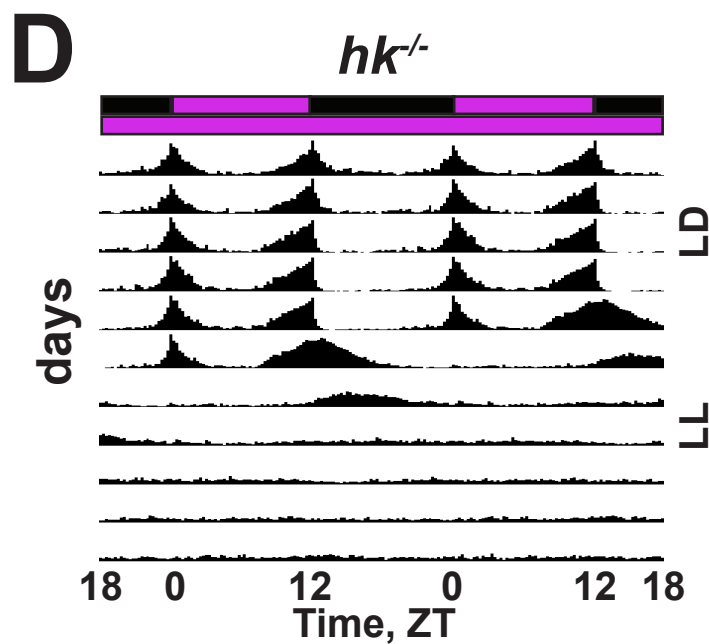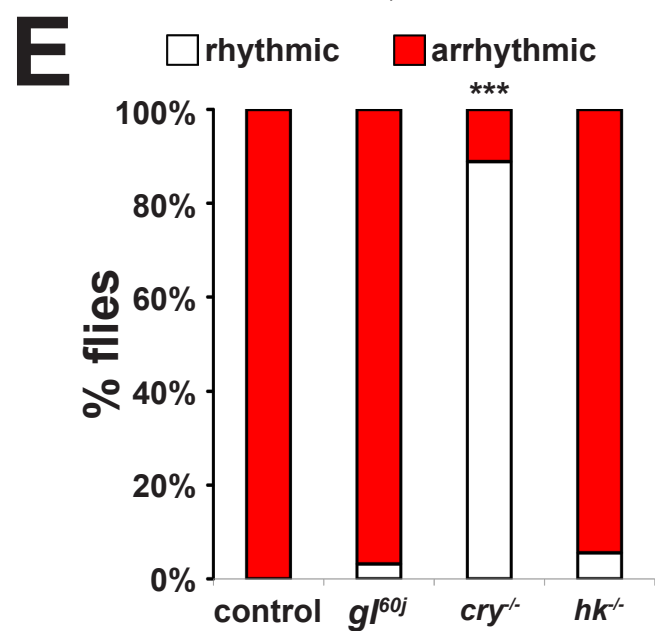

Supplement: S2 Fig — (A-C) Representative double plotted locomotor actogram in 5 days of standard 12h:12h UV (365 nm, 400 μW/cm2) light: dark (LD) followed by 6 days of constant UV light condition (LL). (A) Control (w1118; n = 47 flies) flies have normal entrainment in LD and becomes arrhythmic in LL. (B) glass60j (n = 87 flies) also becomes arrhythmic in LL. (C) cry-/- (n = 87 flies) on the other hand maintain rhythmicity in UV LL. (D) hk-/- (n = 90 flies) become arrhythmic in LL. (E) Percentages of rhythmic and arrhythmic flies in LL. Data are represented as mean ± S.E.M. ***p < 0.001 vs. control. (PDF) [file pone.0201927.s002.pdf]

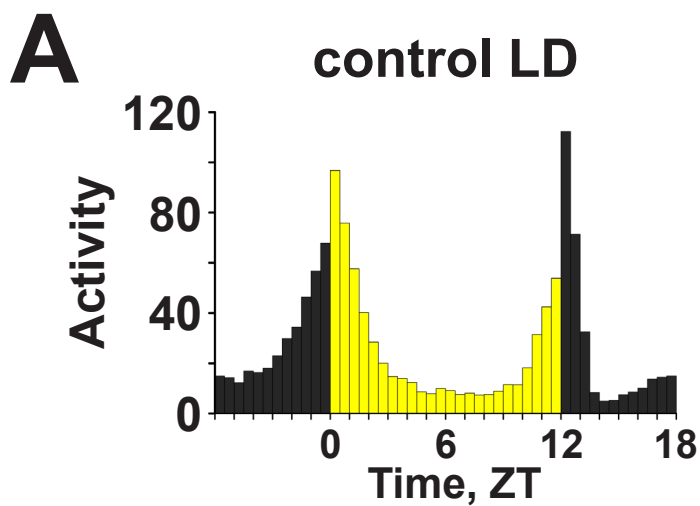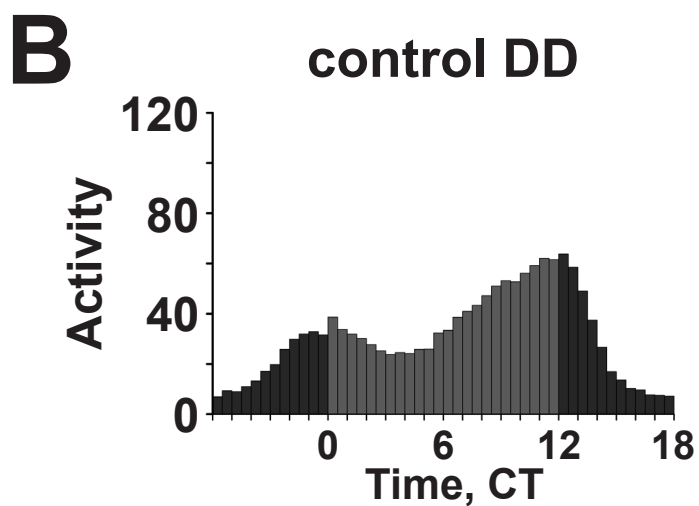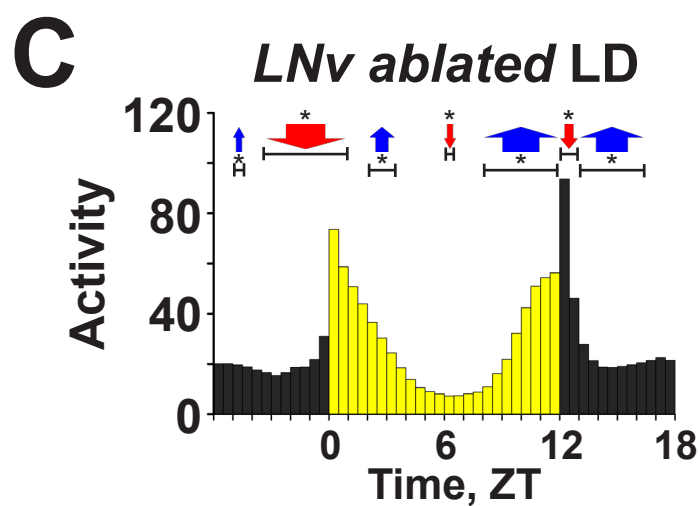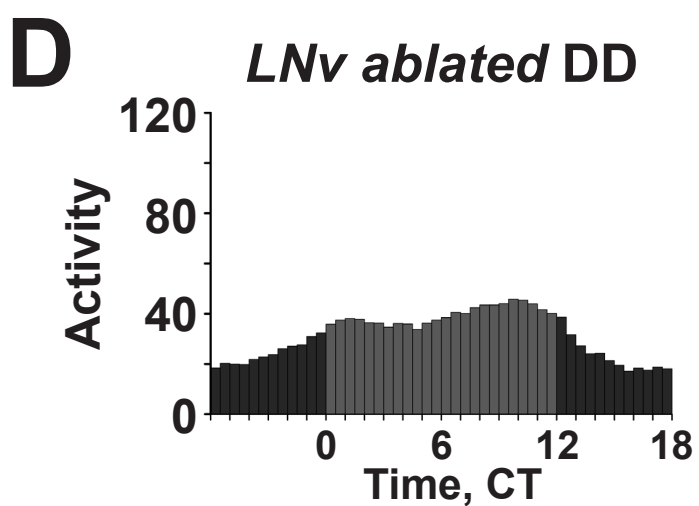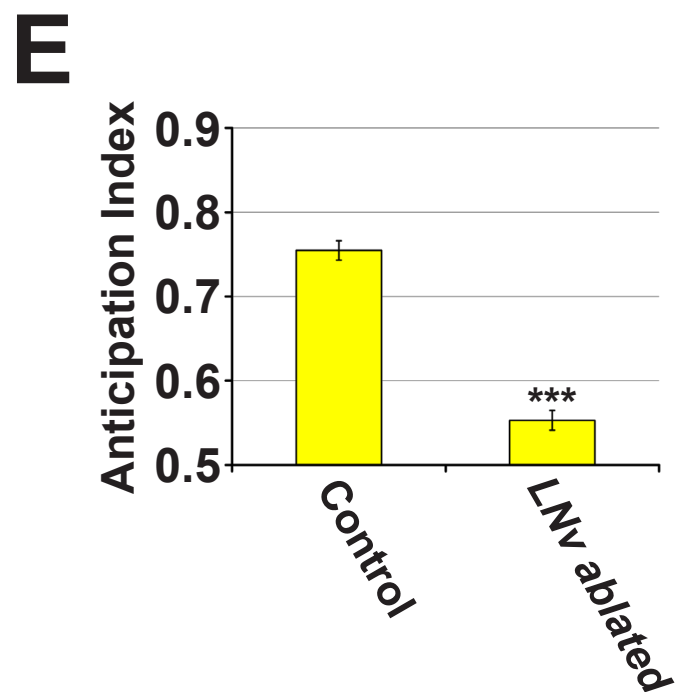

Supplement: S3 Fig — (A-D) Average activity plot of control (n = 96 flies) (top panels) and LNv ablated flies (UAS-hid, rpr; pdfGAL4-p12c; n = 256 flies) (bottom panels) in standard 12h:12h white light: dark (LD) (left panels; 5 days) followed by constant darkness (DD) (right panels; 5 days). Arrows represent significantly higher (blue arrow, *p<0.05) or significantly lower (red arrow, *p<0.05) average activity in LNv ablated flies compared to control in the represented bin(s) throughout the day during LD. Compared to (A) control flies (n = 96 flies), (C) PDF+ (LNv) ablated flies (UAS-hid, rpr; pdfGAL4-p12c; n = 256 flies) show defective locomotor activity in LD. (B, D) Average activity plot in constant darkness (DD) (5 days) that followed LD. (B) Control and (D) LNv ablated flies both maintain rhythmicity in DD, but LNv ablated flies show defective locomotor activity in DD compared to control flies. (E) Harrisingh morning anticipation index for control (left) versus LNv ablated (UAS-hid, rpr; pdfGAL4-p12c; right) during LD. LNv ablated flies have significantly lower morning anticipation compared to control during white light LD (control, n = 64 versus LNv ablated, n = 159, ***p<0.001). Data are represented as mean ± S.E.M. *p < 0.05; ***p < 0.001 vs. control. (PDF) [file pone.0201927.s003.pdf]

**A*****cry*<sup>-/-</sup> vs. *LNv* ablated**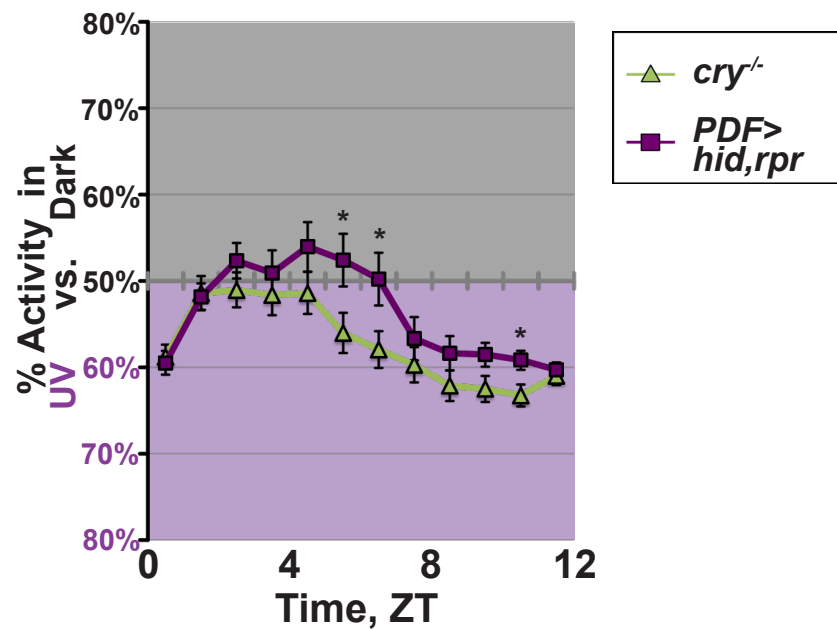**B*****hk*<sup>-/-</sup> vs. *LNv* ablated**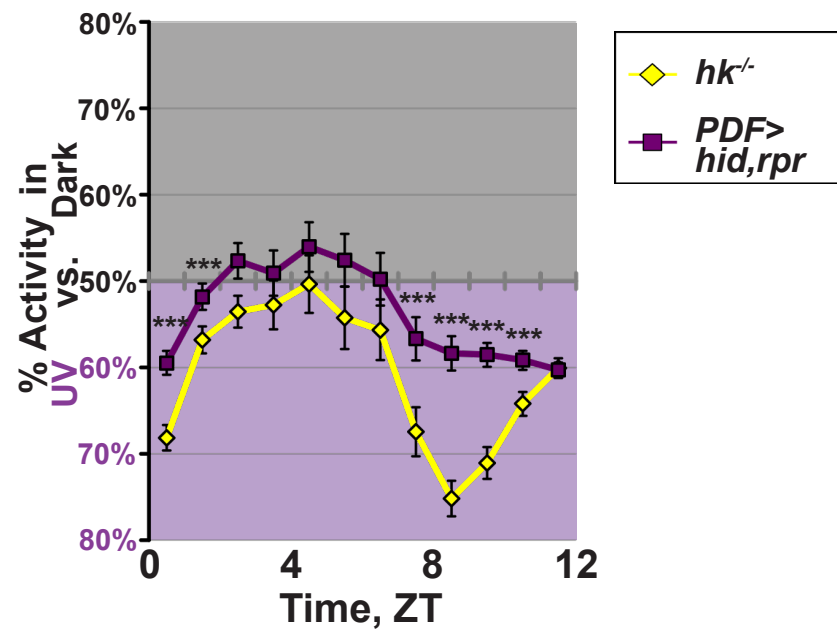

Supplement: S4 Fig — (A-B) UV avoidance behavior measured by preference for shaded environment vs. UV-exposed (365 nm, 400 μW/cm2) calculated by percent of activity in each environment over total activity for each ZT. LNv ablated flies (UAS-hid, rpr; pdfGAL4-p12c; n = 76 flies) closely mimic the time-of-day dependent circadian modulation and valence of UV light avoidance behavior of (A) cry-/- (n = 78, modified from Baik et al., 2017, PNAS) and (B) hk-/- (n = 77, modified from Baik et al., 2017, PNAS) flies. Data are represented as mean ± S.E.M. *p < 0.05; ***p < 0.001. (PDF) [file pone.0201927.s004.pdf]
